# Hepatic Angiomyolipoma: Diagnostic Findings and Management

**DOI:** 10.1155/2012/410781

**Published:** 2012-12-23

**Authors:** Kenya Kamimura, Minoru Nomoto, Yutaka Aoyagi

**Affiliations:** Division of Gastroenterology and Hepatology, Graduate School of Medical and Dental Sciences, Niigata University, Asahimachi 1-757, Chuo-ku, Niigata 951-8122, Japan

## Abstract

Angiomyolipoma (AML) is a benign mesenchymal tumor that is frequently found in the kidney and, rarely, in the liver. The natural history of hepatic AML has not been clarified, and, because of the similar patterns in imaging studies, such as ultrasonography, computed tomography, and magnetic resonance imaging, some of these tumors have been overdiagnosed as hepatocellular carcinoma in the past. With an increase in the number of case reports showing detailed imaging studies and immunohistochemical staining of the tumor with human melanoma black-45, the diagnostic accuracy is also increasing. In this paper, we focused on the role of noninvasive imaging studies and histological diagnosis showing distinctive characteristics of this tumor. In addition, because several reports have described tumor progression in terms of size, recurrence after surgical resection, metastasis to other organs, and portal thrombosis, we summarized these cases for the management and discussed the indications for the surgical treatment of this tumor.

## 1. Introduction

Hepatic angiomyolipoma (HAML) is a rare benign mesenchymal liver tumor first described by Ishak [1] in 1976; it belongs to a group of perivascular epithelioid cell tumors called PEComa [2]. Until date, approximately 300 cases have been reported [3–10]; however, its natural history has not been clarified. The tumor composed of blood vessels, smooth muscle, and adipose cells and due to the variety of predominance of these tissues, its patterns in imaging studies have resulted in a difficulty in diagnosis and misdiagnosis of the tumor as hepatocellular carcinoma (HCC) in some cases [6, 11]. Therefore, the preoperative correct diagnosis has been difficult; however, recent advances in imaging diagnosis through a combination of ultrasonography (US), computed tomography (CT), magnetic resonance imaging (MRI), and angiography and specific immunohistochemical analysis of this tumor using human melanoma black-45 antigen (HMB-45) staining have resulted in accurate diagnosis and it is reported that the current accurate preoperative diagnosis was made in 25%–52% of cases [8, 9]. The majority of these tumors are believed to be clinically benign during a mean follow-up period of 6.8 years [11]; however, an increasing number of cases and aggressive changes including growth in size, recurrence after surgical resection, metastasis, and invasive growth pattern into the parenchyma and along the vessels have been reported. In this paper, we have focused on the characteristic features of this tumor shown in imaging studies and by histological analysis, summarized these cases showing aggressive patterns, and discussed management of the patients and indications for surgical treatment.

## 2. Natural History and Laboratory Findings

The natural history of this tumor has not been clarified. Most cases were found as incidental liver tumors upon health screening or imaging examinations for other diseases. It usually follows a benign clinical course while some patients visited hospitals with unspecific symptoms of abdominal discomfort, fullness, and other such complaints. More than half of the renal AML are considered to be associated with tuberous sclerosis which features the loss of heterozygosity at *TSC1* (9q34) and *TSC2* (16p13), while it is estimated to be 5–15% in the liver [6]. Thus the etiology of most of these tumors in the liver is unknown; most cases have no history of liver diseases or specific symptoms, and no changes in laboratory data are seen. Moreover, serum levels of the tumor markers alpha-fetoprotein, protein induced by vitamin K absence or antagonist II, carcinoembryonic antigen, and carbohydrate antigen 19-9 were normal. 

## 3. Imaging Studies

Since this tumor is composed of various tissues, such as lipomatous, myomatous, and angiomatous tissue in various proportions, the imaging studies show a wide array of features depending on the predominance of each tissue. The tumor showing the predominance of lipomatous tissue is likely to be correctly diagnosed; however, myomatous and angiomatous variant poses diagnostic problems since it is difficult to be distinguished from malignant tumors. 

### 3.1. Ultrasonography (US)

**Figure 1:**
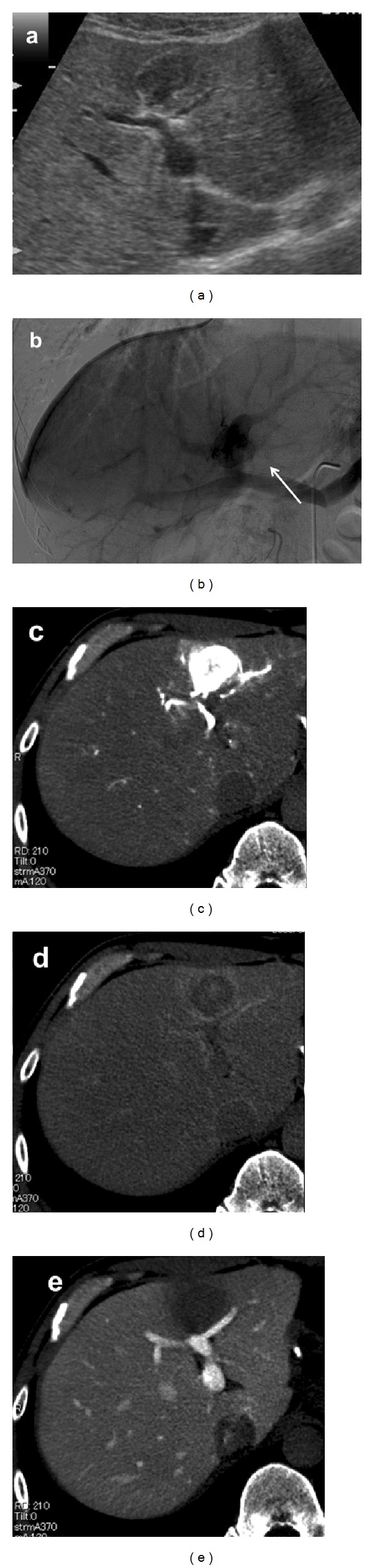
Imaging studies of HAML: (a) ultrasonography and (b) angiography. White arrow indicates the tumor staining in the portal venous phase. (c) 1st phase of CTA, (d) 2nd phase of CTA, and (e) CTAP. ((e) is from [12] with permission from Springer).

US images may vary depending on the tissue components affected by the tumor. High echogenic lesions can be observed because of lipomatous and myomatous tissue, and angiomatous tissue may result in low echoic lesions in tumor images (Figure 1(a)). If the tumor has predominance of lipomatous tissue, the differential diagnosis with hepatic hemangioma is difficult by sonography alone. Color doppler sonography shows punctiform or filiform vascular distribution pattern if the tumor has predominance of angiomatous tissue. Recent reports showed the diagnostic effectiveness of contrast-enhanced US (CEUS) [10, 13]. Li et al. reported that CEUS revealed the typical imaging characteristics of HAML, that is, an inhomogeneous hyperenhancing pattern in the arterial phase and prolonged enhancement during the portal and Kupffer phases. 

### 3.2. Computed Tomography (CT)

Plain CT showed homogeneous or heterogeneous low-density lesions, and contrast-enhanced dynamic CT showed highly enhanced lesions in the arterial phase, prolonged enhancement in the portal phase, and, occasionally, defective lesions in the late venous phase depending on the component of the tumor tissue. A density of less than 20 hounsfield units in plain CT is useful to determine the involvement of lipomatous tissue [14]. The difficulty is, however, to diagnose the tumor that is myomatous tissue and angiomatous variant. For this point, modification of size of region of interest in CT was reported to be effective for accurately diagnose renal angiomyolipoma from renal cell carcinoma [15].

### 3.3. Angiography

Abdominal angiography showed marked tumor staining in the arterial phase, which remained in the portal phase (Figure 1(b)). In some tumors, drainage of the hepatic veins can be observed in the late vascular phase. The first phase of CT during hepatic arteriography showed significant hypervascular lesions in the tumor (Figure 1(c)), and the second phase showed the remains of staining and defective lesions in other areas (Figure 1(d)). CT during arterial portography showed areas of defective tumors (Figure 1(d)) [12]. 

### 3.4. Magnetic Resonance Imaging (MRI)

MRI is considered to be the best modality to determine the components of the tumor. Hyperintensity on the T2-weighted image and hyper- or hypointensity on the T1-weighted image are observed depending on the component of tumor tissue [8–10]. Lipomatous lesions may be determined as hyperintensity on the T1-weighted image; they may also be determined by the chemical shift imaging technique. Contrast-enhanced dynamic MRI using gadolinium or the hepatocyte-specific contrast agent gadolinium-ethoxybenzyl-diethylenetriamine pentaacetic acid showed early enhancement in the arterial phase followed by the prolonged enhancement in the portal phase and defective lesions in the hepatobiliary phase.

## 4. Pathological Findings

**Figure 2:**
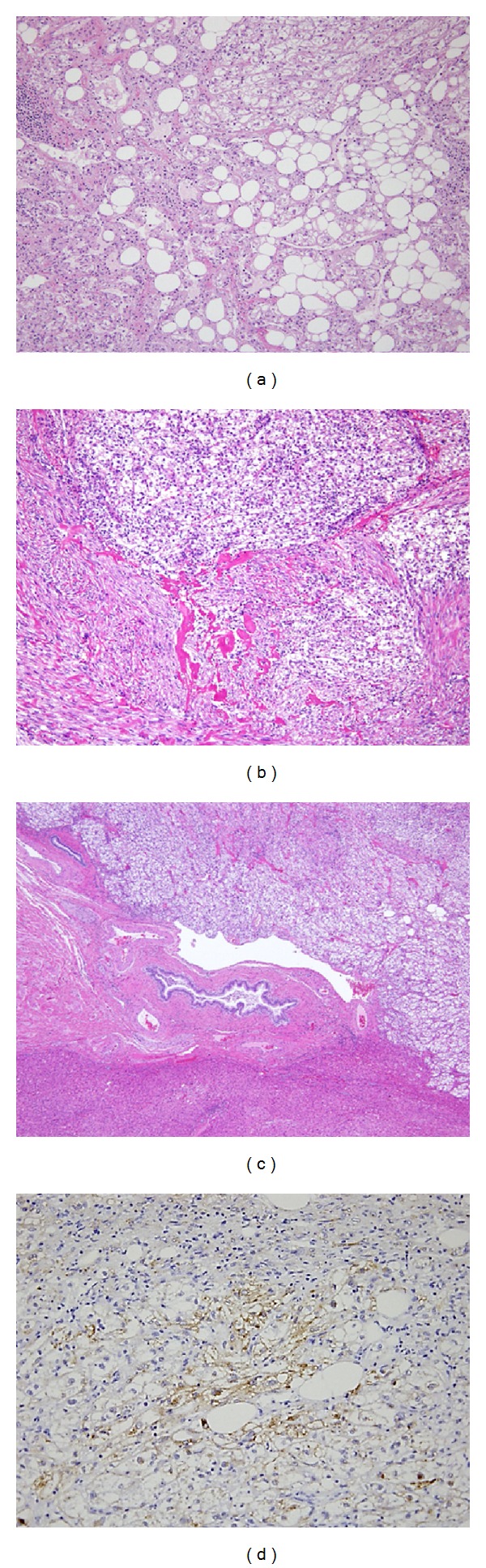
Pathological findings of HAML. The tumor was consisted of lipomatous, myomatous, and angiomatous tissue with variance. (a) A part of the tumor showed predominance of lipomatous tissue and the center of the tumor showed predominance of myomatous and angiomatous tissue (b). (c) The tumor showed aggressive growth pattern infiltrating into the portal vein (HE staining, ×40). (d) Tumor cells were positive for HMB-45 staining (×100) ((a) is from [12] with permission from Springer).

Macroscopic and magnifying glass view of the tumor showed a soft, white to yellow, and well-circumscribed tumor and range in size from 0.1 cm to greater than 36 cm [11]. No signs of chronic inflammation or fibrosis were seen in the surrounding liver tissue. Because HAML comprises lipomatous, myomatous, and angiomatous tissues, microscopic examination showed a mixture of blood vessels, specialized smooth muscle cells, and adipose cells with atypical changes in classic HAML (Figures 2(a) and 2(b)). This variation of mixture levels in one tumor reflected the differences in imaging studies and made accurate diagnosis difficult. It is believed that majority of the HAMLs behave in a benign fashion and even if some cases showed invasive growth pattern into the adjacent hepatic parenchyma, portal triad (Figure 2(c)), and hepatic vein [11]. Tumor cells, especially myomatous cells, were stained positive for HMB-45 in most tumors (Figure 2(d)), for CD34 in the endothelial cells of the blood vessels and for smooth muscle actin in spindle smooth muscle cells. HMB-45 is an antibody that reacts with an oncofetal premelanosome-associated glycoprotein 2, found in neoplastic melanocytes. Also CD63, CD67, desmin, S100, and EMA may also be positive but not specific. These cells were negative for cytokeratin 18, cytokeratin 19, CAM 5.2, hepatocyte paraffin-1. Therefore, the identification of lipomatous, myomatous, and angiomatous tissue by a positive reaction to HMB-45 currently provides the only evidence of HAML [4–6, 18] and can be useful for defining from the other liver tumors such as HCC after the tumor biopsy and surgical resection followed by these immunohistochemical stainings. Although favorable prognosis can be expected for this tumor since the majority of the tumors are benign, however recently, the number of reports of HAML showing malignant potential has increased [3, 5, 8, 9, 12, 16–24] which revealed significant growth, recurrence, metastasis, and poor prognosis summarized in Table 1. Among those cases, Deng et al. reported in their case that HAML showed atypical angiomatous, epithelioid components with pleomorphic and frequent mitosis in the center of the large tumor displayed p53 immunoreactivity, and mutation at exon 7 for p53 and resulted in vascular invasion, distant metastasis, and fatal outcomes [23]. These results indicate that the HMB-45 staining, mitotic analysis by MIB-1 (Ki67), and p53 reactivity following the fine-needle biopsy of the tumor might be useful for the diagnosis of the tumor and its malignant potential.

## 5. Patient Management

Since HAML usually follows benign clinical courses, the majority of the cases can be conservatively treated. However, as not a few cases showed aggressive pattern in their courses [3, 5, 8, 9, 12, 16–24] and because of the low level of accurate diagnoses by imaging studies, and because of the possibility of dissemination of tumor cells into the peritoneal cavity if a tumor is malignant, tumor biopsy has been avoided, and many HAMLs have been surgically resected [3, 8, 9]. With the increasing number of resected samples, careful comparisons of imaging studies and pathological findings have been performed [8, 9, 12, 23]. Therefore, once HAML has been diagnosed by imaging studies, a fine-needle biopsy should be performed to make an accurate diagnosis [19] and to confirm the predominance of the tissue components, the existence of pleomorphic nuclei with high proliferation activity, p53 immunoreactivity, and mutation in p53 if possible. If the aggressive patterns such as vascular invasion, high proliferation of the tumor cells, p53 immunoreactivity were marked [23], or when the imaging findings and biopsy cannot provide a definitive diagnosis, or if the patients have abdominal symptoms, surgical resection should be considered. 

## 6. Discussion

HAML is considered to be a benign mesenchymal tumor [1], and nonsurgical treatment with conservative management involving close followup is therefore suggested for patients with asymptomatic tumors smaller than 5 cm, which have been proved to be a typical HAML by fine-needle aspiration biopsy [3]. As a fact the renal angiomyolipoma often showed perirenal invasion, involvement of the renal vein, and the inferior vena cava, that are not considered as signs of malignant behavior and the angiomatous, epithelioid monophasic or pleomorphic variant might be associated with the aggressive behavior and cellular atypia, mitotic activity, and metastatic lesions are the criteria for the malignant renal angiomyolipoma. However, due to the difficulties in diagnosis by imaging studies, many HAMLs have been surgically resected and have been followed closely for a long period. In addition, by careful analysis of these recent cases [3, 5, 8, 9, 12, 16–24] summarized in Table 1, it was revealed that, although rare, HAMLs may have malignant potential, which may be distinguished by aggressive patterns characterized by (1) significant changes in size in short period; (2) a change of tumor composition (Cases 1, 2, 5, and 6) [9, 16, 17, 19]; (3) metastases to the other organs (Cases 1, 9, and 10) [17, 22, 23]; (4) recurrence after curative surgical resection (Cases 3, 7, 9, 10, 11, 12, and 13) [3, 8, 18, 20, 22–24]; and (5) invasive growth into the vessels (Cases 2, 4, 8, 9, and 10) [12, 17, 21–23]. 

Ohmori et al. reported the first possibly malignant case [16], which showed that a significant increase in the tumor size resulted in liver dysfunction. Fatal progression was observed in nine cases listed by multiple recurrences [3, 8, 18, 20, 22–24] and metastases to the liver, peritoneum, retroperitoneal region, gastrohepatic omentum, pancreas, and lung [17, 22, 23]. The cases reported by Chang [9], Rouquie [21], and us [12] underwent surgical resection, with a suspicious of aggressive patterns and no recurrence has occurred. We also reported that portal thrombosis, that is, high-grade portal vein invasion, found in five cases in Table 1 (Cases 2, 8, 9, 10, and 11) [3, 17, 21–23] may be a clinical marker of the malignant potential and transformation of HAML [12] as it resulted in a significantly aggressive disease and fatal course in 4 cases with multiple recurrences and metastases. This finding might be able to be detected by imaging studies as similar to HCC. In addition, pathological findings of atypical epithelioid component with high proliferation activity, p53 immunoreactivity, and mutation in p53 might be predictive markers for malignant transformation [23]. Based on these reports, as recommended by previous papers [3, 8, 9], long-term follow-up of HAML is necessary after its diagnosis by imaging studies and biopsy specimens and curative surgical treatment. The majority of HAMLs behave as a benign tumors and conservative follow-up may be recommended [25]; however, with increasing number of the reports showing potentially malignant behavior, prompt surgical resection is essential for better prognosis of this tumor.

## 7. Conclusion

We have reviewed noninvasive imaging studies and the role of histological diagnosis showing distinctive characteristics of HAML to increase the rate of accurate diagnoses. In addition, we summarized the cases that showed progressive patterns of the tumor and concluded that a careful followup of the tumor even after the final diagnosis is necessary. We propose that tumor resection is indicated in the following scenarios: (1) the patients show symptoms; (2) the tumor shows an aggressive growth; (3) the tumor shows invasive growth into the vessels evidenced by fine-needle biopsy or imaging studies; (4) the component of the tumor shows atypical epithelioid pattern, high proliferation activity, and/or p53 immunoreactivity; and (5) a definitive diagnosis cannot be made by imaging and pathological studies from malignant tumors. 

## Figures and Tables

**Table 1:** Summary of hepatic angiomyolipoma with progressive growth, recurrence, and metastasis.

Case	Author	Tumor size (cm)	Tumor growth	Resection	Invasive growth	Portal vein thrombus	Recurrence	Metastasis	Prognosis	Clinical features
1	Ohmori et al. [16]	Tumor grew to 18 cm	+	−	+	N/A	−	−	D	Liver dysfunction
2	Dalle et al. [17]	Tumor grew from 15 to 26 cm in 5 years	+	+	+	+	+, 15 cm	+, peritoneum, liver	D	
3	Flemming et al. [18]	0.5 and 10 cm	+	+	+	−	+, 20 cm	+, liver	A	Second resection was performed
4	Kamimura et al. [12]	3.5	+	+	+	+	−	−	A	
5	Rimola et al. [19]	Tumor grew from 6 to 11 in 8 years	+	−	−	N/A	N/A	N/A	A	
6	Chang et al. [9]	Tumor grew from 8 to 27 in a year	+	+	−	−	N/A	−	A	
7	Croquet et al. [20]	19	+	+	N/A	N/A	+, 33 cm	−	A	Second resection was performed
8	Rouquie et al. [21]	9	N/A	+	+	+	−	−	A	
9	Nguyen et al. [22]	11	N/A	+	+	+	+	+, liver, peritoneum, gastrohepatic omentum, and retroperitoneum.	D	
10	Deng et al. [23]	18	N/A	+	+	+	+, 11 cm	+, lung	D	
11	Yang et al. [3]	13	N/A	+	+	+	+	+, liver, lung	D	
12	Parfitt et al. [24]	14	N/A	+	+	N/A*	+	+, trapezius muscle, lung, pancreas	D	
13	Ding et al. [8]	8	N/A	+	N/A	N/A	+	−	D	

N/A: information was not applicable.

D: dead and A: alive.

*Portal vein invasion was found in recurrent tumors.
